# Prévalence du portage asymptomatique du plasmodium chez les donneurs bénévoles de sang à Kisangani, République Démocratique du Congo

**DOI:** 10.11604/pamj.2014.17.320.3778

**Published:** 2014-04-28

**Authors:** Jacques Ossinga Bassandja, Salomon Batina Agasa, Joris Losimba Likwela

**Affiliations:** 1 Département de Médecine Interne, Faculté de Médecine et de Pharmacie, Université de Kisangani, Kisangani, République Démocratie du Congo; 2 Département de Santé Publique, Faculté de Médecine et de Pharmacie, Université de Kisangani, Kisangani, République Démocratie du Congo

**Keywords:** Paludisme, Prévalence parasitaire, Donneur bénévole de sang, Transfusion sanguine, République Démocratique du Congo, malaria, parasite prevalence, blood donor, blood transfusion, Democratic Republic of Congo

## Abstract

**Introduction:**

Le paludisme transfusionnel est une réalité en Afrique Sub-saharienne, en raison des transfusions sanguines répétées, peu ou non contrôlées et où les donneurs sont en majorité potentiellement porteurs d'hématozoaires. L'objectif de cette étude était de déterminer la prévalence du portage asymptomatique du plasmodium chez les donneurs bénévoles de sang à Kisangani.

**Méthodes:**

Une étude transversale a été menée au Centre Provincial de Transfusion Sanguine à Kisangani du 1^er^ Décembre 2012 au 31 Mars 2013 et a concerné 480 donneurs bénévoles de sang.

**Résultats:**

La prévalence du portage asymptomatique du plasmodium chez les donneurs bénévoles de sang était de 28,3%. *Plasmodium falciparum* était l'espèce la plus répandue (96,3%). Près de la moitié des donneurs avait une parasitémie supérieure à 2000 parasites/µl. Les facteurs qui étaient significativement associés à la parasitémie étaient le jeune âge, le 1^er^ don, et la non utilisation de la moustiquaire imprégnée d'insecticide à longue durée (MILD).

**Conclusion:**

Les résultats de cette étude montrent que la prévalence du portage asymptomatique du plasmodium chez les donneurs bénévoles de sang était élevée, constituant ainsi un risque important de transmission du parasite aux receveurs souvent en mauvais état général. Cependant, l'utilisation de la MILD et la fidélisation des donneurs bénévoles semblent constituer des moyens utiles de réduction du risque de portage asymptomatique du Plasmodium. Une sensibilisation et éventuellement des distributions ciblées de MILD aux donneurs, en particuliers les plus jeunes, pourraient réduire considérablement le portage du Plasmodium parmi les donneurs de sang et ainsi réduire le risque de paludisme transfusionnel.

## Introduction

La transfusion sanguine peut sauver des vies, mais peut également transmettre des infections virales, bactériennes et parasitaires potentiellement mortelles [1]. Il a été estimée qu'environ 40% (13 million d'unités) de sang donné dans les pays en développement ne font pas l'objet d'un dépistage de principales infections transmissibles par le sang [2]. Tandis que des efforts considérables ont été réalisés ces dernières années pour la réduction du risque de transmission des maladies qu'on ne sait pas encore soigner comme l'hépatite B, l'hépatite C, le SIDA, on s'inquiète peu des maladies infectieuses curables comme le paludisme [3, 4].

Le paludisme transfusionnel est une réalité en Afrique Sub-saharienne, en raison des transfusions sanguines répétées, peu ou non contrôlées et où les donneurs sont en majorité potentiellement porteurs (asymptomatiques) d'hématozoaires. En zone d'endémie, la prévalence des donneurs de sang impaludés varie selon les régions et peut aller jusqu’à 55%, ce qui traduit un risque potentiel de transmission du parasite par cette voie [4–13].

En RD Congo (RDC) en général et dans la ville de Kisangani en particulier, peu de données sont disponibles sur le risque de paludisme transfusionnel. C'est dans ce cadre que la présente étude se propose de déterminer la prévalence du portage asymptomatique du plasmodium chez les donneurs bénévoles de sang à Kisangani.

## Méthodes

Cette étude a été réalisée au Centre Provincial de Transfusion Sanguine (CPTS) de la Province Orientale à Kisangani. Le CPTS constitue la référence en matière de production des composants sanguins pour la région. Il s'agissait d'une étude transversale s′étendant du 1er Décembre 2012 au 31 Mars 2013.

Pendant la période de l’étude, au total 997 candidats au don de sang se sont présentés et ont fait l'objet d'une sélection clinique et biologique pour être retenu pour le don de sang selon les critères de sélection fixés par le Centre National de Transfusion Sanguine (CNTS) [14]. A l'issu de cette sélection, 480 sujets ont été retenu et constituent les sujets de la présente étude. Les fiches ad hoc utilisées par le Programme National de Transfusion Sanguine ainsi que les registres de laboratoire ont été passés en revu afin de recueillir les variables d'intérêt, en l'occurrence, l’âge, le sexe, les résultats de la goutte épaisse, de la densité parasitaire et de l'espèce plasmodiale. Ces données ont été complétées par une interview pour renseigner sur les mesures de prévention antipaludiques utilisées par les sujets.

Chaque échantillon de sang a servi à confectionner, sur une même lame, un frottis mince et une goutte épaisse colorés par le Giemsa. L′espèce plasmodiale était identifiée sur le frottis, la densité parasitaire sur la goutte épaisse, selon la formule retenue par l′Organisation Mondiale de la Santé. La parasitémie était appréciée en comptant les parasites asexués pour 200 leucocytes, soit une évaluation pour un microlitre de sang (tablant sur une moyenne de 8 000 leucocytes par microlitre). Lorsque le nombre de parasites comptés était inférieur à 10 pour 200 leucocytes, la numération était effectuée par rapport à 500 leucocytes. La parasitémie était exprimée par microlitre.

Les analyses de laboratoire ont été effectuées par un technicien de laboratoire formé par le Programme National de Lutte contre le Paludisme (PNLP) sur la microscopie de la goutte épaisse, dont la remise à niveau se fait régulièrement lors de différentes études d'efficacité des antipaludiques au niveau du site sentinelle de Kisangani. Le contrôle de qualité a été réalisé au Laboratoire Provincial de la Santé Publique de Kisangani (LPSP).

Les données recueillies étaient regroupées et présentées à travers les tableaux de fréquences. Pour la comparaison des proportions, nous avons eu recours au chi carré de Pearson à un seuil de signification de 0,05. Lors que les conditions de réalisation du test de chi carrée n’étaient pas satisfaites, nous avons eu recours au test exact de Fischer. Les analyses ont été réalisées à l'aide du logiciel Epi Info 3.5.3.

## Résultats

Les caractéristiques des enquêtés, leur statut de donneurs (ancien ou nouveau) et leurs habitudes relatives à la prévention du paludisme sont présentées dans le Tableau 1. Il apparait que la majorité des donneurs étaient jeunes, de sexe masculin et anciens donneurs bénévoles de sang. Quant au recours aux mesures préventives, 90,6% affirmaient utiliser la moustiquaire imprégnée d'insecticide.

**Tableau 1:** Caractéristiques des donneurs de sang, leur statut de donneurs (ancien ou nouveau) et leurs habitudes relatives à la prévention du paludisme

Caractéristiques	n	%
**Age**		
18-27	243	50,6
28-37	129	26,9
38-47	83	17,2
48-57	57	11,9
58-plus	6	1,2
**Sexe**		
M	461	96
F	19	4
**Statut de donneur**		
Anciens	454	94,6
Nouveaux	26	5,4
**Utilisation de la MILD**		
Oui	435	90,6
Non	45	9,4

Le Tableau 2 montre la prévalence parasitaire chez les donneurs bénévoles de sang, la densité parasitaire et les espèces plasmodiales rencontrées chez les donneurs de sang. Sur 480 enquêtés, 136 étaient porteurs d'hématozoaires dans le sang, soit une prévalence de 28,3%. La densité parasitaire est très variable, cependant près de 1 donneur sur 2 avait une parasitémie supérieure à 2000 parasites/µl. La lecture du frottis mince indique que *Plasmodium falciparum* était la principale espèce rencontrée.

**Tableau 2:** Prévalence parasitaire, densité parasitaire et espèces plasmodiales rencontrées chez les donneurs de sang

	N	%
**Goutte épaisse**		
Négatif	344	71,7
Positif	136	28,3
**Parasitémie**		
< 1000 parasites/µl	10	7,4
1000 – 2000 parasites/µl	60	44,1
> 2000 parasites/µl	66	48,5
**Espèce plasmodiale**		
Plasmodium falciparum	131	96,3
Plasmodium malariae	4	2,9
Plasmodium ovale	1	0,8

Le Tableau 3 présente l'analyse de la prévalence parasitaire en fonction des caractéristiques sociodémographiques des sujets, leur statut de donneurs (ancien ou nouveau) et leurs habitudes relatives à la prévention du paludisme.

**Tableau 3:** Analyse de la prévalence parasitaire en fonction des caractéristiques sociodémographiques des sujets, leur statut de donneurs (ancien ou nouveau) et leurs habitudes relatives à la prévention du paludisme

Caractéristiques	n	% de GE +	0R (IC95%)	P-val
**Age**				<0,001*
18-27	243	(119) 49,0	1	
28-37	129	(12) 9,3	0,19 (0,11- 0,33)	
38-47	83	(2) 2,4	0,05 (0,01- 0,19)	
48-57	57	(2) 3,5	0,07 (0,02- 0,28)	
58-plus	6	(1) 16,7	0,34 (0,06- 2,05)	
**Sexe**				0,84**
M	461	(131) 28,4	1	
F	19	(5) 26,3	0,93 (0,43- 1,99)	
**Statut de donneur**				<0,001**
Anciens	454	(120) 26,4	1	
Nouveaux	26	(16) 61,5	2,33 (1,66- 3,27)	
**Utilisation des MILD**				<0,001**
Oui	435	22,3	1	
Non	45	86,7	3,89 (3,15- 4,79)	

* Test eact de Fisher

** Chi carré de Pearson

Les facteurs qui étaient significativement associés à la parasitémie étaient le jeune âge, le 1^er^ don, et la non utilisation de la moustiquaire imprégnée d'insecticide. Le sexe des donneurs ne semblaient pas influencer le portage du parasite.

## Discussion

Pendant la période de l’étude, sur 480 donneurs de sang, 28,3% de sujets étaient porteurs du plasmodium, parmi lesquels 96% de *P falciparum*. La densité parasitaire était variable, cependant près de la moitié de donneurs porteurs du parasite avaient une densité qui dépassait 2000 parasites/µl.

Une revue de la littérature sur le risque de transmission du paludisme par la transfusion sanguine en Afrique subsaharienne sur une période allant de 1980 à 2009 avait montré que la prévalence parasitaire chez les donneurs de sang variait considérablement de 0,1% au Kenya à 55% au Nigeria [15]. Les prévalences les plus élevées ont été rapportées en Afrique de l'Ouest, en l'occurrence au Nigeria et au Bénin [4, 11, 15]. Ailleurs, une prévalence moins élevée était observée, en particulier en Afrique de l'Est et Australe [9, 15]. Les principales sources de variations sont essentiellement, le niveau d'endémicité dans le milieu et la saison au moment de l’étude. A Kisangani, le paludisme est à transmission intense et pérenne. Ce qui pourrait expliquer la prévalence élevée observée qui est compatible avec la prévalence estimé en 2007 sur le sang prelevé au cours de l'enquête démographique et de santé qui a révélé une prévalence moyenne de 33,5% en RDC [16]. Ceci indique un risque important de paludisme transfusionnel justifiant d'attirer l'attention des décideurs. Ce d'autant plus que l'espèce retrouvée est principalement le *P. falciparum*, espèce responsable des formes graves de la maladie.

Il convient de relever, qu’à ce jour, les documents de politique nationale de transfusion sanguine, soit sont muets au sujet du paludisme transfusionnel, soit recommandent de tester tout sang donné par l'examen de la goutte épaisse, laissant penser, sans le dire clairement, que le sang des donneurs positifs à la goutte épaisse devrait être rejeté [14, 17]. Cette recommandation est réitérée au cours des sessions de formations pendant lesquels il est demandé aux prestataires de rejeter les poches de sang de donneurs porteurs du plasmodium. En pratique, cette recommandation est respectée par le CPTS de Kisangani, mais dans les hôpitaux généraux de référence de la ville, le sang prélevé des donneurs familiaux est administré aux receveurs à qui une cure complète de traitement antipaludique de 1^ère^ ligne est ensuite administrée. Ces tergiversations des dispositions réglementaires ont été relevées dans la littérature. Dans certains cas, il est demandé de faire systématiquement le dépistage du plasmodium, d'autres recommandent un rejet du sang infecté par le plasmodium, d'autres encore de mettre les patients recevant un sang infecté sous un traitement antipaludique, d'autres d'ajouter un antipaludique au sang afin d’éradiquer le plasmodium in vitro, tandis que certains demandent de mettre tous les receveurs sous un traitement antipaludique présomptif en région endémique [15].

Dans la présente étude, la parasitémie était significativement associés au jeune âge des donneurs. Dans la population générale, la prévalence parasitaire en RDC était plutôt significativement élevée chez les sujets les plus âgés [16]. Cependant, l'observation faite chez les donneurs dans notre étude va dans le même sens que la plupart des études africaines qui retrouvait chez les donneurs de sang une prévalence plus élevé chez les sujets jeunes [4, 11]. Les donneurs bénévoles fidélisés présentaient un risque de parasitémie significativement moindre. Diop S et coll. avaient observé au Sénégal, que le caractère irrégulier des dons était associé à un portage asymptomatique du *Plasmodium* [8].

L'utilisation de la MILD était rapportée par la plupart des donneurs de sang. Les donneurs de sang qui n'utilisaient pas la MILD avait un risque de portage asymptomatique du Plasmodium près de 4 fois plus que ceux qui rapportait son utilisation. Si l'on considère que la MILD peux réduire de 45% la survenu du paludisme sévère [18], alors elle peut être considérer comme un moyen efficace à la fois pour réduire la demande en produits sanguins et pour augmenter la disponibilité en produits sanguins de qualité via la réduction du risque de portage chez les donneurs.

## Conclusion

Les résultats de cette étude montrent que la prévalence du paludisme chez les donneurs bénévoles de sang était élevée, constituant ainsi un risque important de transmission du parasite aux receveurs souvent en mauvais état général. Cependant, l'utilisation de la MILD et la fidélisation des donneurs bénévoles semblent constituer des moyens utiles de réduction du risque de portage asymptomatique du Plasmodium. Une sensibilisation et éventuellement des distributions ciblées de MILD aux donneurs, en particuliers les plus jeunes, pourraient réduire considérablement le portage du Plasmodium parmi les donneurs de sang et ainsi réduire le risque de paludisme transfusionnel.
